# Supplementary material for: A multi-sample approach increases the accuracy of transcript assembly
Source: Nat Commun. 2019 Nov 1;10:5000. doi: 10.1038/s41467-019-12990-0 (PMC6825223; doi:10.1038/s41467-019-12990-0)
Supplement: Supplementary file 1 — Supplementary Information [file 41467_2019_12990_MOESM1_ESM.pdf]

“A MULTI-SAMPLE APPROACH INCREASES THE ACCURACY OF TRANSCRIPT ASSEMBLY”

by Song et al.

SUPPLEMENTARY INFORMATION FOR THE MANUSCRIPT:

"A MULTI-SAMPLE APPROACH INCREASES THE ACCURACY OF TRANSCRIPT ASSEMBLY"

Li Song, Sarven Sabunciyan, Guangyu Yang and Liliana Florea

Supplementary Methods

Previous work (extended)

Genome-based transcript assembly is a central problem in transcriptomics, and entails piecing together the RNA-seq read alignments to infer the exon-intron structure of the expressed genes and transcripts along the genome. Multiple transcript assembly methods were reported since the revolutionary introduction of RNA sequencing [1]. Virtually all of these methods use a two step approach, first building a graph representation of a gene and its splice variants from RNA-seq read alignments, and then traversing the graph to select a subset of transcripts that are likely present in the sample (see [2] and references therein). To build a representative structure for the gene, Cufflinks [3] uses an overlap graph in which reads are vertices and two reads (read pairs) are connected if they overlap and have compatible splice patterns. Scripture [4] and IsoLasso [5] build connectivity graphs, in which the set of vertices consists of all genomic positions, and two positions are connected by an edge if they are adjacent in the genome or are the endpoints of an intron. More recent tools, including iReckon [6], CLASS [7], CLASS2 [8], CIDANE [9], StringTie [10] and Scallop [11] have preferentially employed splice or subexon graphs due to their compactness and intuitive nature. In such representations, exons (subexons) are vertices and edges are introns connecting the exons. (In a subexon graph, consecutive subexons within an exon are additionally connected.) Lastly, TransComb [12] employs a junction graph, where the nodes represent edges in an initial splicing graph and edges connect two incident edges in the splicing graph. While these diverse data structures can encode similar sets of transcripts, step 2, transcript selection, is determinant for the program's accuracy performance. For instance, Cufflinks' minimum partition algorithm selects a mathematically minimum number of transcripts, which limits the number of splice isoforms that can be reported. Several methods, including IsoLasso, SLIDE, iReckon and CIDANE, employ 'best fit' linear-programming or expectation maximization-based approaches to select a subset of transcripts that optimize an objective function, often with a regularization penalty to reduce the number of transcripts reported. Other popular approaches include dynamic programming optimization to solve a SET_COVER problem for the set of transcripts and splice patterns (constraints) (CLASS2), network flow optimization algorithms (Traph [13], StringTie, FlipFlop [14]), 'combing' for a PATH_COVER in a weighted junction graph (TransComb), or iteratively decomposing a splice graph into phase-preserving paths, namely paths that can be uniquely associated with one transcript, with linear programming algorithms (Scallop).

All of the above methods generate a set of partial transcripts (transfrags) for a given RNA-seq samples. Since most experiments involve multiple RNA-seq samples, transfrags from all samples are further 'merged' into more complete exon-intron structures to determine a consensus set of transcripts, or *meta-annotations*. Existing meta-assemblers include Cuffmerge, included with the Cufflinks package, StringTie-merge from the StringTie package, and more recently TACO [15]. TACO builds a 'path' graph from the input transcripts, where a 'path' is a sequence of consecutive splice junctions (partial transcript) represented as a vertex and two vertices are connected if they have compatible junction patterns, and iteratively selects the most abundant paths (isoforms). Despite the importance of meta-annotations for subsequent quantification and differential gene expression analyses, however, there has been relatively little effort in designing mathematically rigorous meta-assemblers. Lastly, only a small number of studies have focused on simultaneous multi-sample transcript assembly. These include CLIIQ [16], an early protoype algorithm that uses an integer linear programing (ILP) approach with variables the full set of isoforms; MiTie [17], which builds a splicing graph representing the gene and maximizes a likelihood function using mixed integer programming with a regularization penalty; and ISP [18], which solves an LP or ILP problem iteratively on a weighted connectivity graph derived from the input samples. While marking significant conceptual advances, they scale poorly (MiTie) or otherwise have limited performance in detecting splicing variation (ISP), as demonstrated in [19].

Supplementary Figures

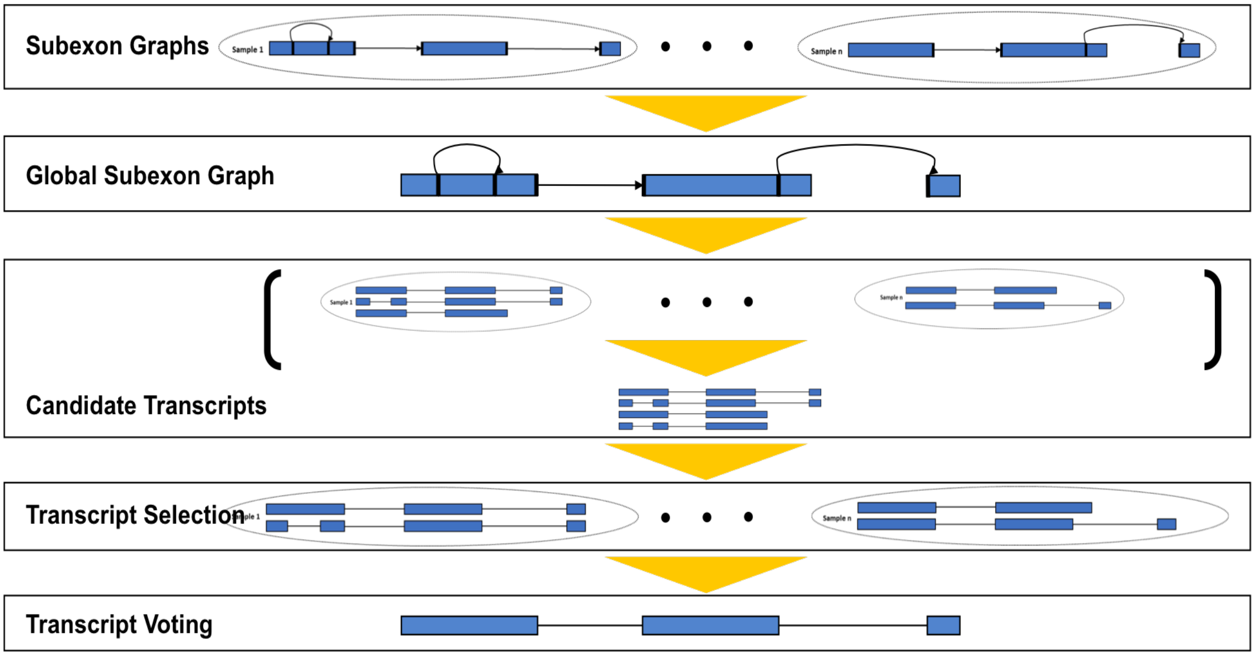

Supplementary Figure 1. Overview of the PsiCLASS algorithm. *Step 1. Build sample-level subexon graphs from aligned reads and splice reads.* PsiCLASS builds a subexon graph for each sample by clustering overlapping read alignments into regions, dividing regions into subexons at splice junctions (inferred from spliced reads), and connecting with edges subexons that are adjacent within the same region or connected by an intron. *Step 2. Build and refine a global subexon graph*, by merging sample-level subexon graphs and employing intron and subexon filters that evaluate information simultaneously *across all* samples. *Step 3. Enumerate or select a set of candidate transcripts* using dynamic programming across all samples. *Step 4. Select a subset of transcripts in each sample*, using a greedy strategy that iteratively select an optimal transcript (with global subexon graph-based dynamic programming). *Step 5. Select a unified set of meta-annotations* from among the sample-level transcripts, with voting.

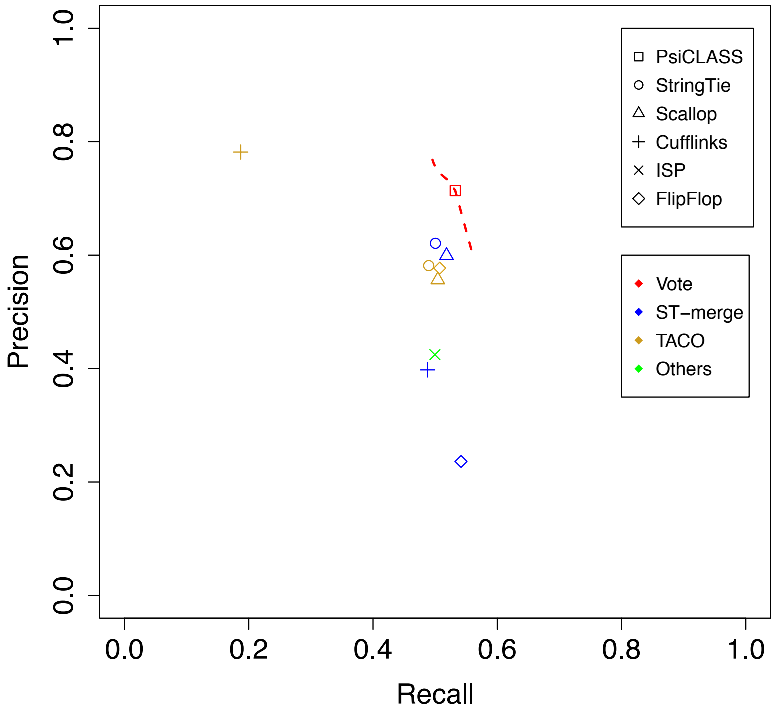

Supplementary Figure 2. Performance evaluation of PsiCLASS and existing reference methods at the level of meta-annotations on simulated data. Methods tested include combinations of three single-sample assemblers (Cufflinks, StringTie and Scallop) and two meta-assemblers (TACO and StringTie(ST)-merge), and two multi-sample integrated methods (ISP and FlipFlop), where TACO and ST-merge were used to aggregate the outputs from individual samples into a unified set of meta-annotations. Below, the shape of the point represents the single-sample assembly tool used, and the color represents the aggregation method. For PsiCLASS, the red curve shows the variation in performance as the weighted voting cutoff varies among 0, 1, 2, 4, 8, 16 (right to left). PsiCLASS produces the highest precision and its sensitivity is comparable with the best of the other methods. *Recall* = TP/(TP+FN), *Precision* = TP/(TP+FP). Source data are provided as a Source Data file.

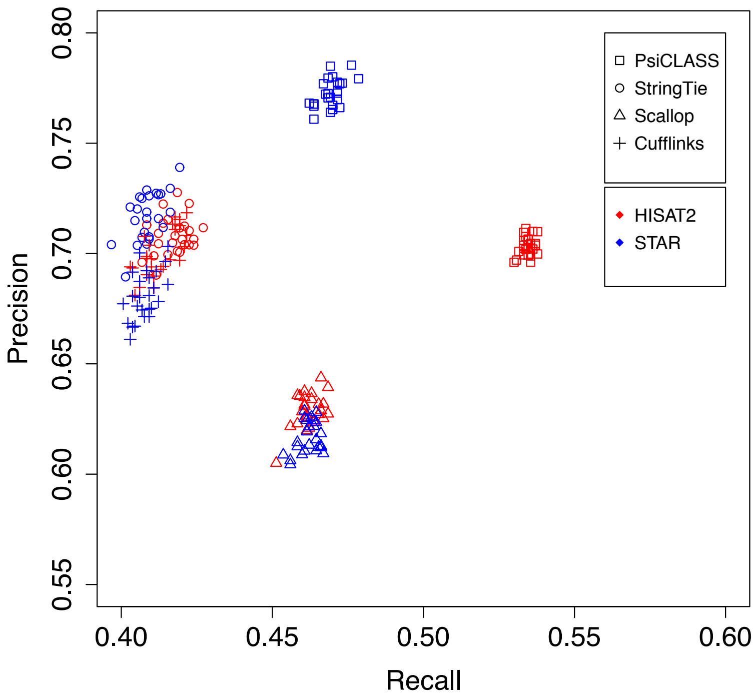

Supplementary Figure 3. Comparison of transcript assembly methods at the sample-level and for different alignment tools. Each point represents the performance of the stated method on one of the 25 simulated samples. The shape of the mark represents the transcript assembly method (StringTie, Scallop and PsiCLASS), and the color indicates the RNA-seq alignment tool (Hisat2 and STAR). All methods perform similarly with the two alignment methods, with Hisat2 leading to a slight increase in performance. When assembly methods are compared, PsiCLASS (with Hisat2) using a global subexon graph leads to improved accuracy at sample level, with the highest per sample average recall, 28% higher than StringTie and 16% higher than Scallop, and precision comparable to StringTie and Scallop.

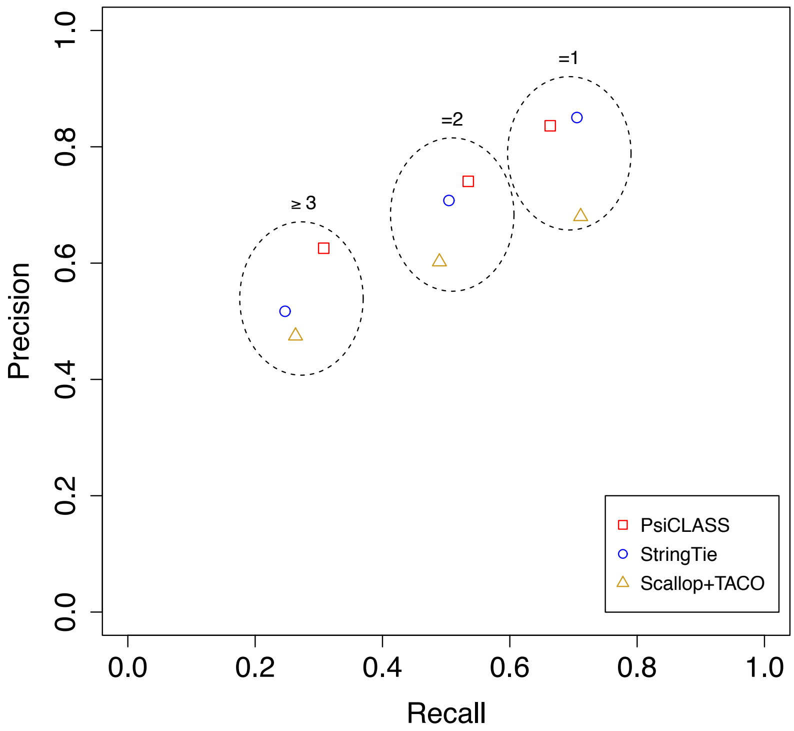

Supplementary Figure 4. Performance of methods on simulated data based on gene splicing complexity. Genes were divided into low (1 transcript/gene; 680 genes, 680 transcripts), medium (2 transcripts/gene; 166 genes, 332 transcripts) and high (3 or more transcripts/gene; 106 genes, 431 transcripts) complexity and methods were evaluated for each group. PsiCLASS is the best performer on the high and medium complexity genes (two leftmost groups), whereas StringTie has the best overall performance on the low complexity group, followed closely by PsiCLASS. Source data are provided as a Source Data file.

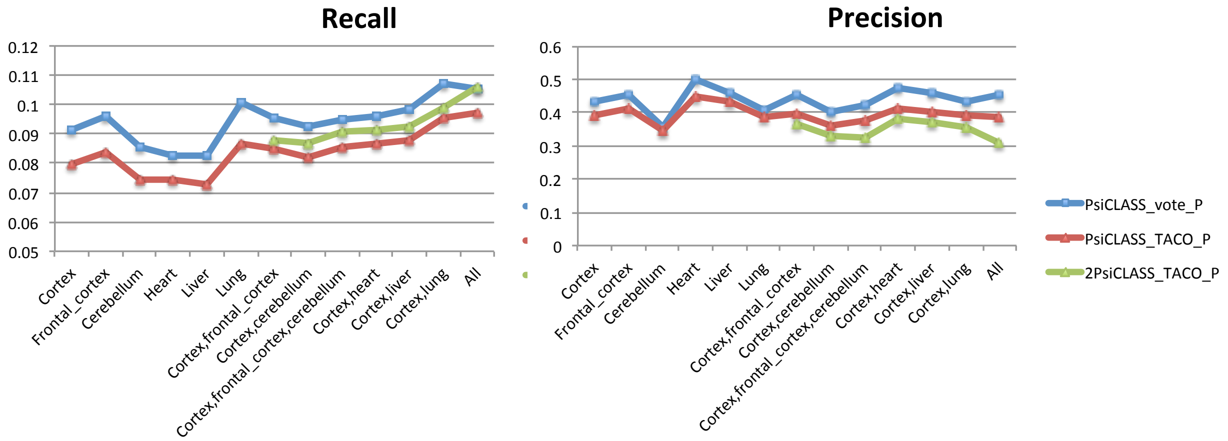

Supplementary Figure 5. Performance of PsiCLASS with heterogeneous collections of data. Three different methods were tested for their ability to generate meta-annotations from single-, two- and multi-condition experiments, using GTEx RNA-seq data from six tissues (cortex, frontal cortex, cerebellum, heart, liver and lung): *i)* PsiCLASS with voting on all samples in a single and/or multi-tissue collection; *ii)* PsiCLASS with TACO on all samples in a single and/or multi-tissue collection; and *iii)* PsiCLASS on all samples of a given tissue, followed by meta-assembly between tissue collections with TACO. Source data are provided as a Source Data file.

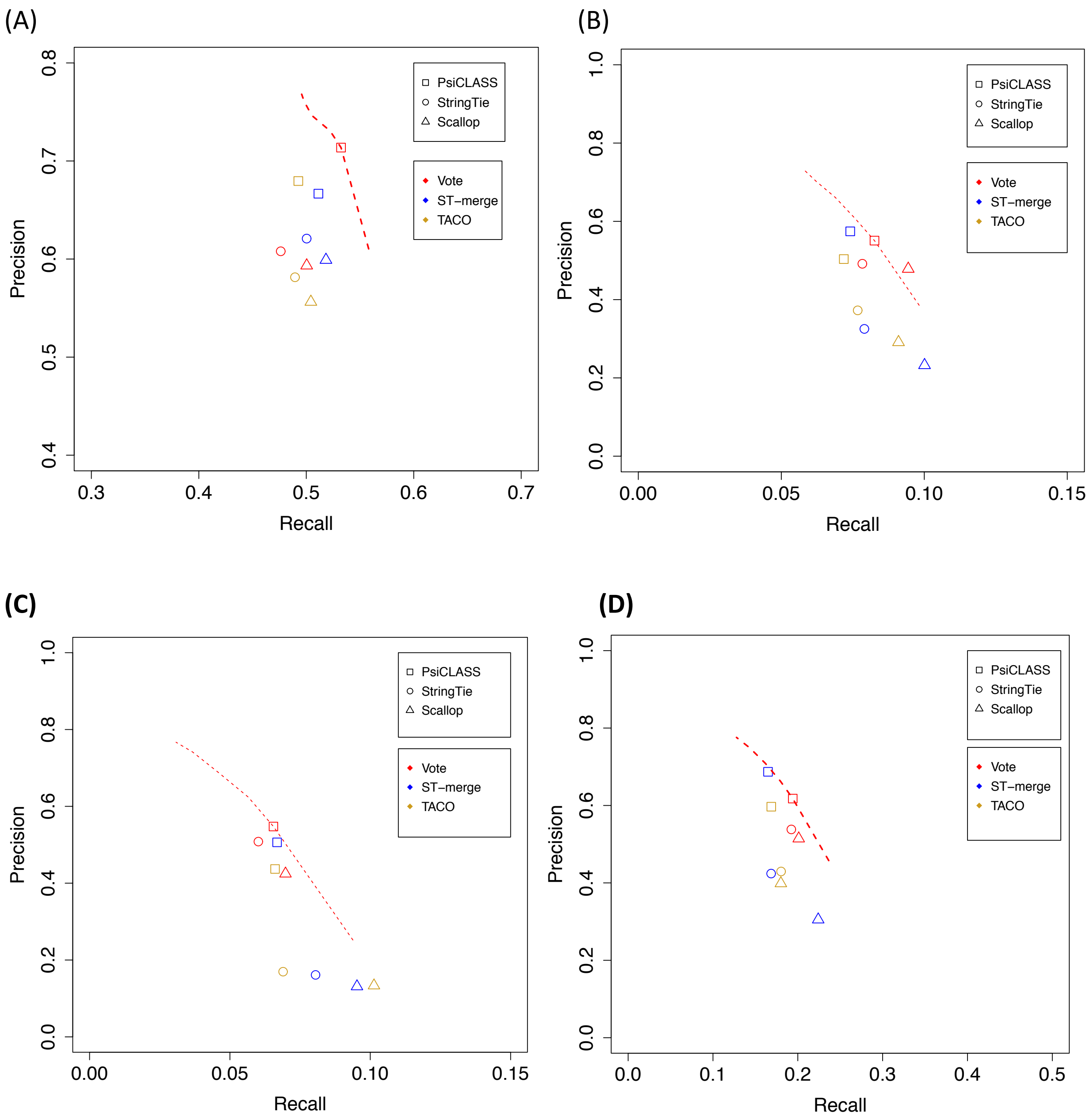

Supplementary Figure 6. Performance evaluation of combination methods on simulated and real data: (A) all method combinations, meta-annotations, simulated data (25 samples); (B) all method combinations, meta-annotations, Geuvadis data (25 samples); (C) all method combinations, meta-annotations, total RNA from human liver (73 samples); and (D) all method combinations, meta-annotations, mouse hippocampus samples, healthy and with induced epileptic seizures (44 samples). For PsiCLASS, the weighted voting cutoff is varied among 0, 1 (default), 2, 4, 8, 16 (shown right-to-left, as red curves). The default cutoff (1.0) was used when voting was applied to all other programs. PsiCLASS's performance is robust with the aggregation method. Also, voting drastically improves over traditional aggregation methods (TACO, ST-merge) for all single-sample assemblers. Source data are provided as a Source Data file.

Supplementary Figure 7 (A).

(A)

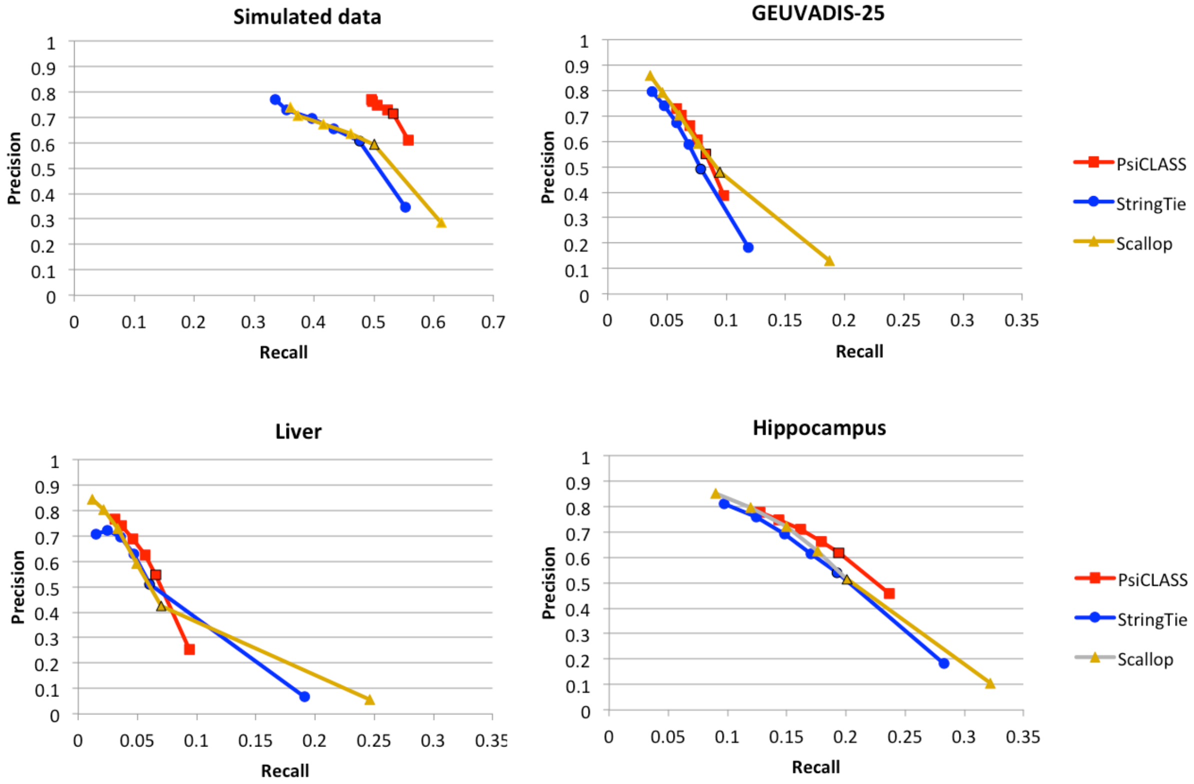

(B)

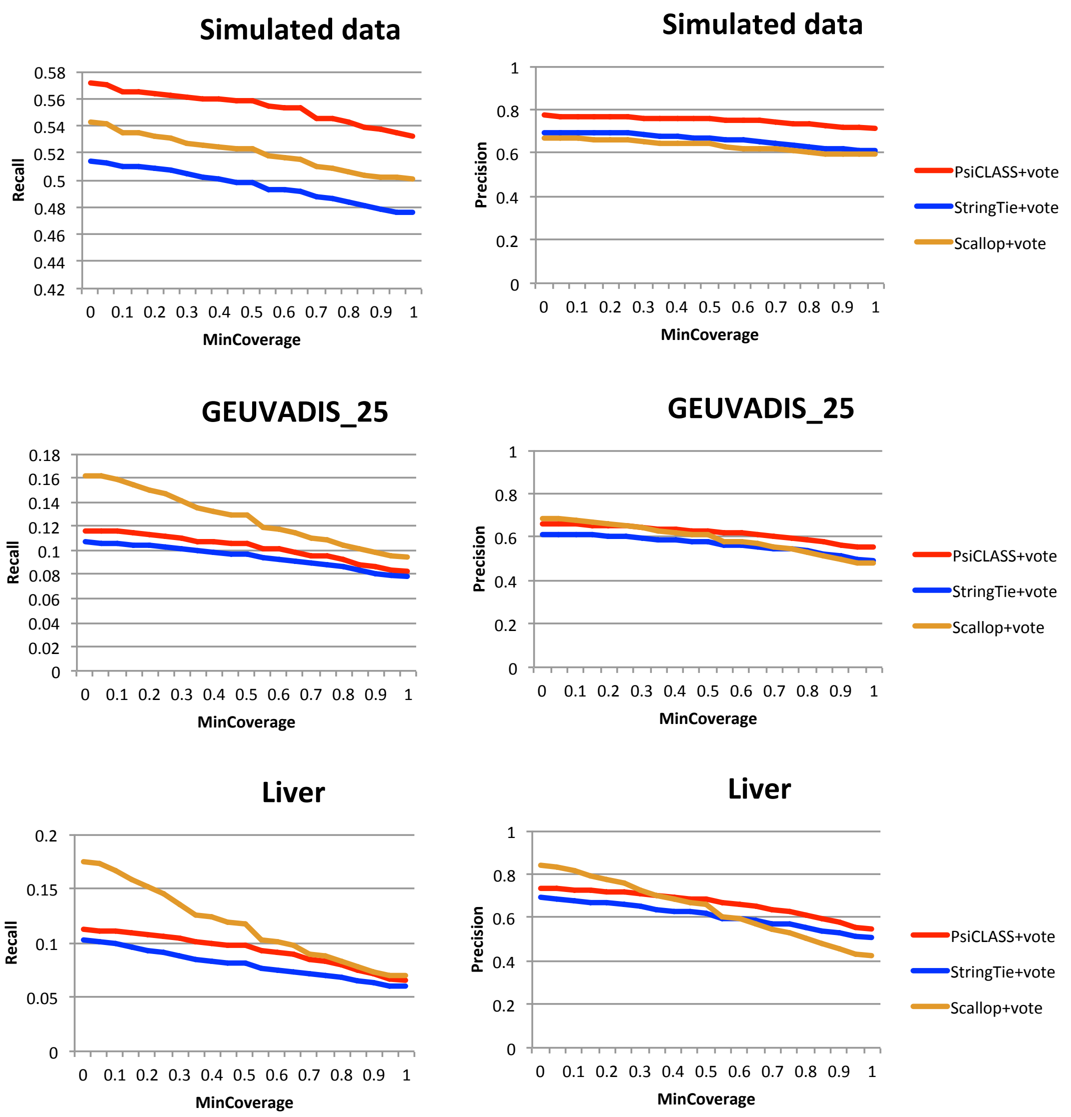

Supplementary Figure 7. Effect of voting - performance at *full-length transcript level* (A) and *partial transcript-level* (B), for the three single-sample assemblers with the voting aggregation method. *(A)* Performance when the weighted voting cutoff varies (0, 1, 2, 4, 8, 16; right-to-left). PsiCLASS maintains a slight edge in full-length reconstructed transcripts over the other single-sample assemblers for cutoff values >=1.0 (default). When no filter is used (cutoff 0.0; union set of transcripts), StringTie and Scallop have significantly higher sensitivity, however at a sharp drop in precision, with PsiCLASS offering the best tradeoff. *(B)* Recall and precision when requiring a minimum transcript coverage fraction (*MinCoverage*), for the default voting parameter (1.0). A predicted transcript is deemed to match a reference transcript at coverage cutoff *c* if its intron chain is a sub-chain containing more than (or equal to) a fraction *c* of the reference transcript's introns. Source data are provided as a Source Data file.

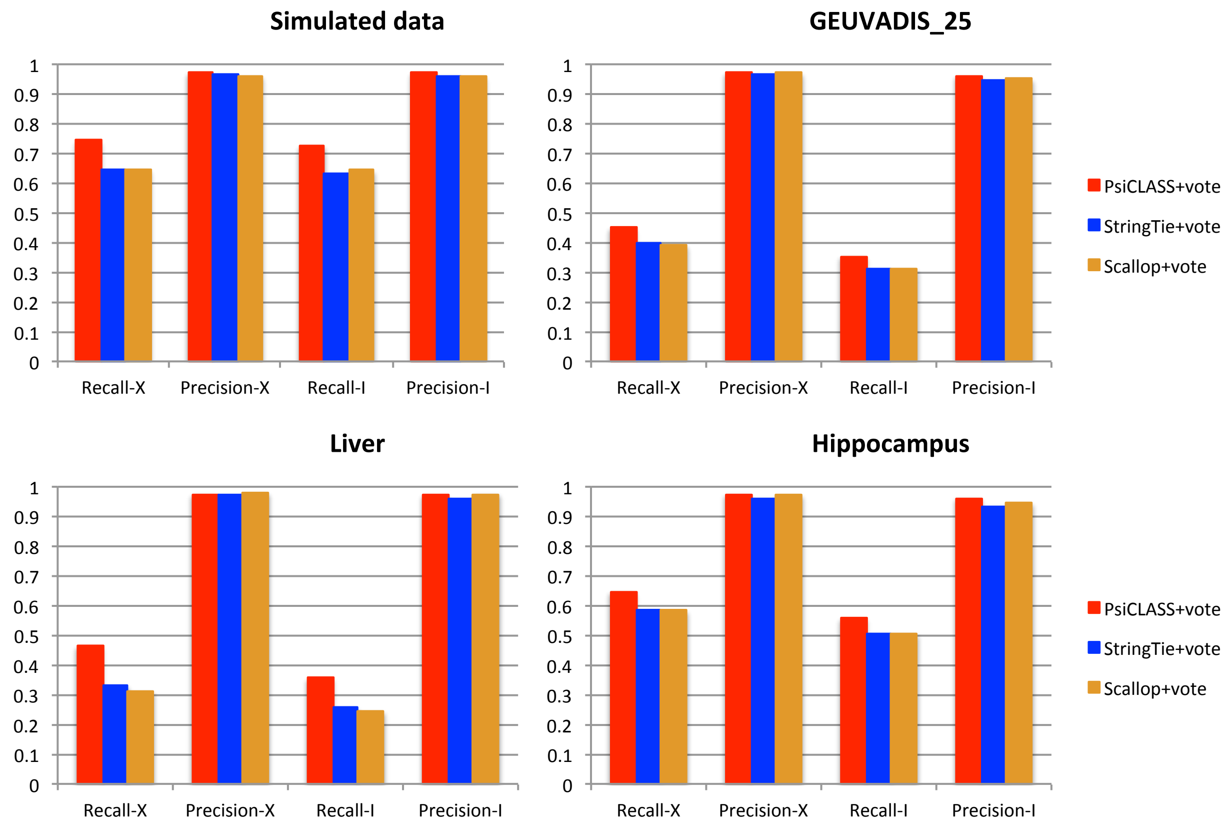

Supplementary Figure 8. Effect of feature selection - performance at *intron* and *internal exon* level for the three single-sample assemblers with the voting aggregation method. PsiCLASS captures 10-40% more introns, and 10-50% more internal exons. Source data are provided as a Source Data file.

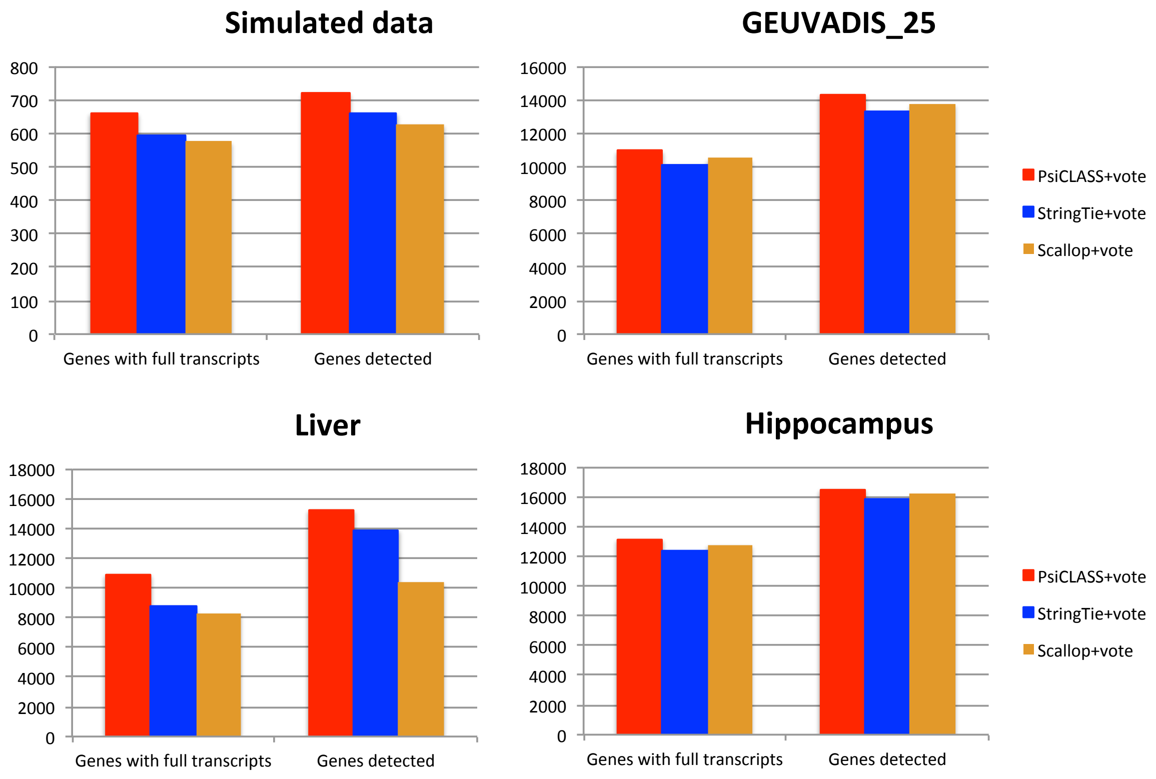

Supplementary Figure 9. Effect of shared subexon graph – performance in terms of completeness of gene model, for the three single-sample assemblers with the voting aggregation method. PsiCLASS builds more complete gene models, as reflected in the number of (reference) genes with full-length transcripts (7-25% more than StringTie and 3-31% more than Scallop), and captures more known (reference) genes (4-10% more than StringTie and 2-46% more than Scallop), as classified by Cuffcompare against the reference set of annotations for each experiment. Source data are provided as a Source Data file.

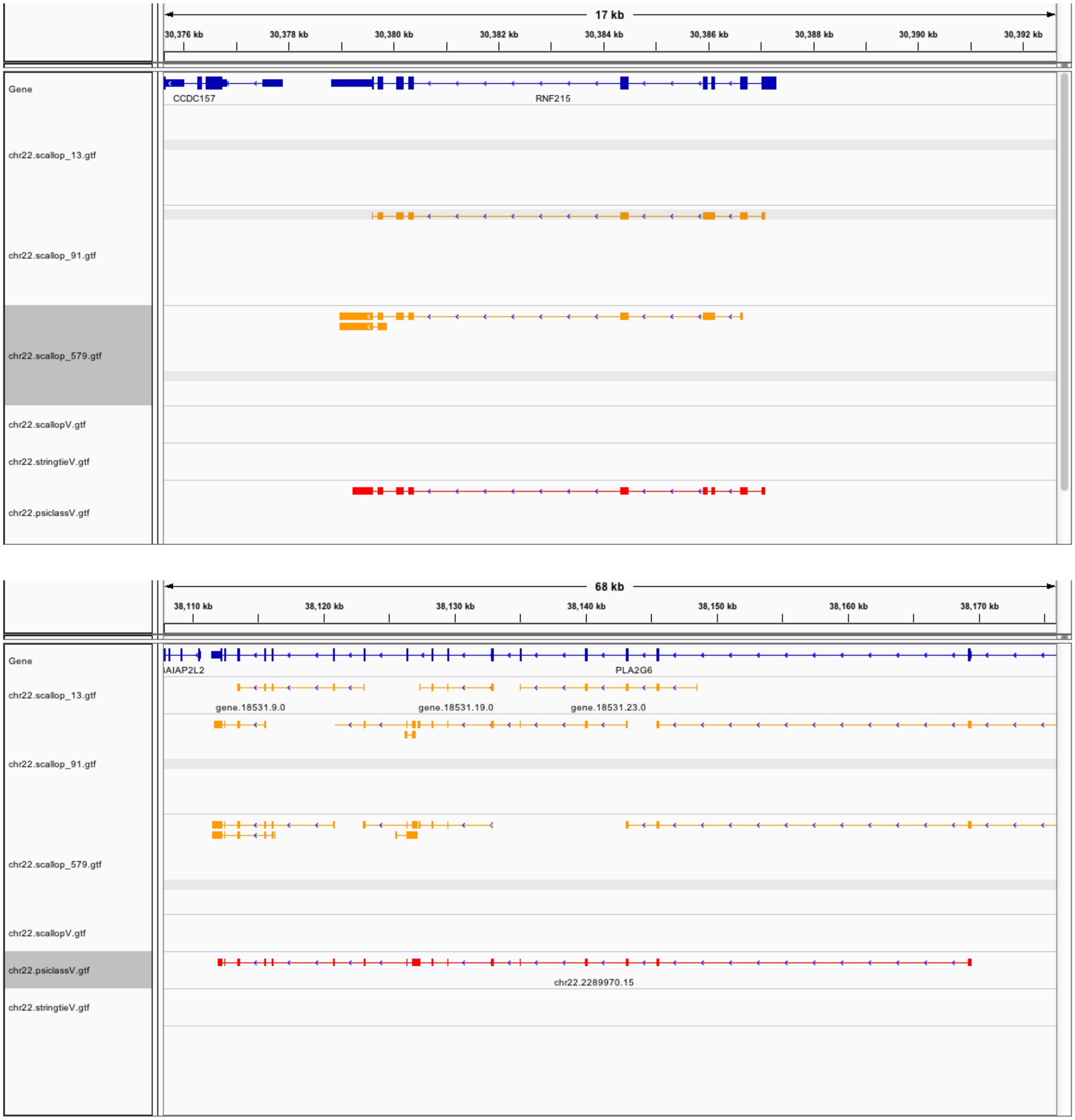

Supplementary Figure 10. Effect of shared subexon graph – completeness of gene model (examples). PsiCLASS (red) predicts full-length transcripts at the RNF215 (top) and partial transcripts at the PLA2G6 (bottom) gene loci in the liver RNA-seq collection, whereas both StringTie and Scallop miss the genes, when the voting aggregation method is used with all three tools. (Panels include sample-level Scallop predictions in 3 randomly selected samples showing partial reconstructions.) RNF215 and PLA2GA are expressed at low levels in liver (www.proteinatlas.org).

REFERENCES

1. Wang, Z., Gerstein, M. & Snyder, M. RNA-Seq: a revolutionary tool for transcriptomics. *Nat Rev Genet* **10**, 57-63 (2009).
2. Canzar, S. & Florea, L. Computational methods for transcript assembly from RNA-seq reads. in *Computational Methods for Next Generation Sequencing Data Analysis*, Vol. 1 (ed. Zelikovsky, I.M.a.A.) 199-216 (Wiley-Interscience, John Wiley and Sons, Inc., Hoboken, NJ, 2016).
3. Trapnell, C. *et al.* Transcript assembly and quantification by RNA-Seq reveals unannotated transcripts and isoform switching during cell differentiation. *Nat Biotechnol* **28**, 511-5 (2009).
4. Guttman, M. *et al.* Ab initio reconstruction of cell type-specific transcriptomes in mouse reveals the conserved multi-exonic structure of lincRNAs. *Nat Biotechnol* **28**, 503-10 (2010).
5. Li, W., Feng, J. & Jiang, T. IsoLasso: a LASSO regression approach to RNA-Seq based transcriptome assembly. *J Comput Biol* **18**, 1693-707 (2011).
6. Mezlini, A.M. *et al.* iReckon: Simultaneous isoform discovery and abundance estimation from RNA-seq data. *Genome Res* **23**, 519-29 (2013).
7. Song, L. & Florea, L. CLASS: constrained transcript assembly of RNA-seq reads. *BMC Bioinformatics* **14 Suppl 5**, S14 (2013).
8. Song, L., Sabunciyan, S. & Florea, L. CLASS2: accurate and efficient splice variant annotation from RNA-seq reads. *Nucleic Acids Res* **44**, e98 (2016).
9. Canzar, S., Andreotti, S., Weese, D., Reinert, K. & Klau, G.W. CIDANE: comprehensive isoform discovery and abundance estimation. *Genome Biol* **17**, 16 (2016).
10. Pertea, M. *et al.* StringTie enables improved reconstruction of a transcriptome from RNA-seq reads. *Nat Biotechnol* **33**, 290-5 (2015).
11. Shao, M. & Kingsford, C. Accurate assembly of transcripts through phase-preserving graph decomposition. *Nat Biotechnol* **35**, 1167-1169 (2017).
12. Liu, J., Yu, T., Jiang, T. & Li, G. TransComb: genome-guided transcriptome assembly via combing junctions in splicing graphs. *Genome Biol* **17**, 213 (2016).
13. Tomescu, A.I., Kuosmanen, A., Rizzi, R. & Makinen, V. A novel min-cost flow method for estimating transcript expression with RNA-Seq. *BMC Bioinformatics* **14 Suppl 5**, S15 (2013).
14. Bernard, E., Jacob, L., Mairal, J. & Vert, J.P. Efficient RNA isoform identification and quantification from RNA-Seq data with network flows. *Bioinformatics* **30**, 2447-55 (2014).
15. Niknafs, Y.S., Pandian, B., Iyer, H.K., Chinnaiyan, A.M. & Iyer, M.K. TACO produces robust multisample transcriptome assemblies from RNA-seq. *Nat Methods* **14**, 68-70 (2017).
16. Lin, Y.-Y. *et al.* CLIIQ: Accurate comparative detection and quantification of expressed isoforms in a population. *Algorithms in Bioinformatics, LNCS* **7534**, 178–189 (2012).
17. Behr, J. *et al.* MITIE: Simultaneous RNA-Seq-based transcript identification and quantification in multiple samples. *Bioinformatics* **29**, 2529-38 (2013).

18. Tasnim, M., Ma, S., Yang, E.W., Jiang, T. & Li, W. Accurate inference of isoforms from multiple sample RNA-Seq data. *BMC Genomics* **16 Suppl 2**, S15 (2015).
19. Yang, G. & Florea, L. JULiP: An efficient model for accurate intron selection from multiple RNA-seq samples. *2016 IEEE 6th International Conference on Computational Advances in Bio and Medical Sciences (ICCABS)* **00**, 1-6; 'Best Paper' Award (2016).
