# Supplementary material for: A multi-sample approach increases the accuracy of transcript assembly
Source: Nat Commun. 2019 Nov 1;10:5000. doi: 10.1038/s41467-019-12990-0 (PMC6825223; doi:10.1038/s41467-019-12990-0)
Supplement: Supplementary file 3 — Reporting Summary [file 41467_2019_12990_MOESM3_ESM.pdf]

nature research

Corresponding author(s): Liliana Florea

Last updated by author(s): 10/11/2019

Reporting Summary

Nature Research wishes to improve the reproducibility of the work that we publish. This form provides structure for consistency and transparency in reporting. For further information on Nature Research policies, see Authors & Referees and the Editorial Policy Checklist .

Statistics

For all statistical analyses, confirm that the following items are present in the figure legend, table legend, main text, or Methods section.

n/a	Confirmed	
☒	☐	The exact sample size (n) for each experimental group/condition, given as a discrete number and unit of measurement
☒	☐	A statement on whether measurements were taken from distinct samples or whether the same sample was measured repeatedly
☒	☐	The statistical test(s) used AND whether they are one- or two-sided<br>*Only common tests should be described solely by name; describe more complex techniques in the Methods section.*
☒	☐	A description of all covariates tested
☒	☐	A description of any assumptions or corrections, such as tests of normality and adjustment for multiple comparisons
☒	☐	A full description of the statistical parameters including central tendency (e.g. means) or other basic estimates (e.g. regression coefficient) AND variation (e.g. standard deviation) or associated estimates of uncertainty (e.g. confidence intervals)
☒	☐	For null hypothesis testing, the test statistic (e.g. F, t, r) with confidence intervals, effect sizes, degrees of freedom and P value noted<br>*Give P values as exact values whenever suitable.*
☒	☐	For Bayesian analysis, information on the choice of priors and Markov chain Monte Carlo settings
☒	☐	For hierarchical and complex designs, identification of the appropriate level for tests and full reporting of outcomes
☒	☐	Estimates of effect sizes (e.g. Cohen's d, Pearson's r), indicating how they were calculated

Our web collection on statistics for biologists contains articles on many of the points above.

Software and code

Policy information about availability of computer code

Data collection	Raw sequencing data were downloaded from public repositories (SRA). No preprocessing of data was performed.
Data analysis	The PsiCLASS software, which is described in the manuscript, is available free of charge and under the GNU GPL license from GitHub (http://github.com/splicebox/PsiCLASS). All scripts used in the evaluation are provided from the project site in GitHub.

For manuscripts utilizing custom algorithms or software that are central to the research but not yet described in published literature, software must be made available to editors/reviewers. We strongly encourage code deposition in a community repository (e.g. GitHub). See the Nature Research guidelines for submitting code & software for further information.

Data

Policy information about availability of data

All manuscripts must include a data availability statement. This statement should provide the following information, where applicable:

- Accession codes, unique identifiers, or web links for publicly available datasets
- A list of figures that have associated raw data
- A description of any restrictions on data availability

Data Availability
Raw sequence data for the GEUVADIS project can be obtained from ArrayExpress (Accession:E-GEUV-6 [https://www.ebi.ac.uk/arrayexpress/experiments/E-GEUV-6/]), mouse hippocampus data from GenBank (ProjectID:PRJEB18790 [https://www.ncbi.nlm.nih.gov/bioproject/?term=PRJEB18790]), and alignments of simulated data from Zenodo (DOI: 10.5281/zenodo.1407759) [https://zenodo.org/record/1407759]. The liver data has been deposited in GenBank under accession PRJNA575230 [https://www.ncbi.nlm.nih.gov/bioproject/PRJNA575230]. Additionally, the full set of evaluation results represented in Figure 1, Tables 1 and 2, and Supplementary Figures 2, 4-9 are provided as a Source Data file, and scripts for performing the evaluation and links to the assembled transcripts can be obtained from the project site in GitHub. All other relevant data are available upon request.

Field-specific reporting

Please select the one below that is the best fit for your research. If you are not sure, read the appropriate sections before making your selection.

- [x] Life sciences
- [ ] Behavioural & social sciences
- [ ] Ecological, evolutionary & environmental sciences

For a reference copy of the document with all sections, see nature.com/documents/nr-reporting-summary-flat.pdf

Life sciences study design

All studies must disclose on these points even when the disclosure is negative.

Sample size	The manuscript describes a computational method, no animal or biological samples were involved. The number of RNA-seq data samples used for software evaluation were typical of current RNA-seq experiment sizes.
Data exclusions	Randomly selected subsets of RNA-seq samples were used for some experiments, to mimic program behavior with typical and/or varying experiment sizes, for the accuracy and scalability tests.
Replication	Not applicable, each RNA-seq experiment is unique.
Randomization	Not applicable, the software is applied to full collections of data.
Blinding	Not applicable, no grouping was performed.

Reporting for specific materials, systems and methods

We require information from authors about some types of materials, experimental systems and methods used in many studies. Here, indicate whether each material, system or method listed is relevant to your study. If you are not sure if a list item applies to your research, read the appropriate section before selecting a response.

Materials & experimental systems

n/a	Involved in the study	
[x]	[ ]	Antibodies
[x]	[ ]	Eukaryotic cell lines
[x]	[ ]	Palaeontology
[x]	[ ]	Animals and other organisms
[x]	[ ]	Human research participants
[x]	[ ]	Clinical data

Methods

n/a	Involved in the study	
[x]	[ ]	ChIP-seq
[x]	[ ]	Flow cytometry
[x]	[ ]	MRI-based neuroimaging
